# Associations of *IL-2*, *IL-6* and *IL-10* Gene Polymorphisms with Biochemical Parameters and Calcineurin Inhibitor Dosing in Kidney Transplant Recipients

**DOI:** 10.3390/biomedicines14081858

**Published:** 2026-08-18

**Authors:** Anna Bogacz, Paweł Szakoła, Monika Kuciak, Jerzy Sieńko, Maciej Kotowski, Grażyna Kurzawińska, Wojciech Łabędź, Aleksandra E. Mrozikiewicz, Agata Urbaniak, Piotr Olbromski, Dorota Formanowicz

**Affiliations:** 1 Department of Cancer Immunology, Poznan University of Medical Sciences, Rokietnicka 8, 60-806 Poznan, Poland; mkuciak@ump.edu.pl; 2 Department of General, Transplant and Liver Surgery, Pomeranian Medical University, 71-455 Szczecin, Poland; pawel.szakola@pum.edu.pl; 3 Institute of Physical Culture Sciences, Pomeranian Medical University, 70-453 Szczecin, Poland; jsien@poczta.onet.pl; 4 Department of General Surgery and Transplantation, Pomeranian Medical University, 70-111 Szczecin, Poland; maciej.j.kotowski@gmail.com; 5 Laboratory of Molecular Biology, Department of Perinatology, Poznan University of Medical Sciences, Polna 33, 60-535 Poznan, Poland; gkurzawinska@ump.edu.pl; 6 Department of Adult Spine Orthopedics, Poznan University of Medical Sciences, 61-545 Poznan, Poland; wlabedz@ump.edu.pl; 7 Department of Diagnostics and Treatment of Infertility, Poznan University of Medical Sciences, Polna 33, 61-701 Poznan, Poland; a.mrozikiewicz@gmail.com; 8 Department of Perinatology, Poznan University of Medical Sciences, Polna 33, 60-535 Poznan, Poland; a.urbaniak4545@gmail.com; 9 Department of Obstetrics and Gynecology, District Public Hospital in Poznan, Juraszów 7/19, 60-479 Poznan, Poland; olbromski.piotr@gmail.com; 10 Department of Medical Chemistry and Laboratory Medicine, Poznan University of Medical Sciences, Rokietnicka 8, 61-701 Poznan, Poland

**Keywords:** kidney transplantation, graft rejection, cytokine gene polymorphisms, immunosuppressive therapy, biochemical parameters, personalized medicine

## Abstract

**Background/Objectives**: Kidney graft dysfunction remains a major challenge limiting long-term transplant survival despite advances in immunosuppressive therapy. Cytokine signaling pathways have been implicated in the regulation of alloimmune responses and chronic inflammation, but their contribution to long-term graft function and immunosuppressant pharmacokinetics remains incompletely understood. This study investigated associations among selected cytokine gene polymorphisms (*IL-2* −330T>G, *IL-6* −174G>C, and *IL-10* −1082A>G), biochemical and hematological parameters, and calcineurin inhibitor (tacrolimus and cyclosporine) dosing in kidney transplant recipients. **Methods**: A total of 394 kidney transplant recipients receiving tacrolimus or cyclosporine were enrolled. Genotyping of *IL-2* rs2069762, *IL-6* rs1800795 and *IL-10* rs1800896 was performed using real-time PCR. Clinical, biochemical, and pharmacokinetic data were analyzed using univariate and multivariate statistical models adjusted for relevant demographic and clinical covariates. **Results**: *IL-2* rs2069762 and *IL-6* rs1800795 polymorphisms were not associated with clinically relevant differences in biochemical parameters, calcineurin inhibitor dosing, or transplant outcomes. For *IL-10* rs1800896, no statistically significant associations were observed with cyclosporine A blood levels, dose, or C/D ratio. Among tacrolimus-treated patients, the regression model for the C/D ratio was borderline significant (F(2,207) = 3.048, *p* = 0.050, R^2^ = 0.029). However, the genotype-specific association for the heterozygous GA genotype versus AA was not statistically significant in the unadjusted model (B = −0.462, 95% CI: −0.97 to 0.04, *p* = 0.072) or after adjustment for age, sex, and time since transplantation (B = −0.450, 95% CI: −0.95 to 0.05, *p* = 0.078). Although univariate analyses identified associations between *IL-10* rs1800896 and selected biochemical parameters, these findings were not confirmed in multivariable models. No significant associations were observed between the analyzed cytokine polymorphisms and graft rejection, graft survival, patient survival, or clinically relevant markers of kidney graft function. **Conclusions**: The results suggest a possible, although not statistically significant, association between IL-10 rs1800896 and tacrolimus exposure, as assessed by the C/D ratio. This observation should be considered preliminary and interpreted with caution. The lack of *CYP3A5* genotyping and functional assessment of IL-10 activity, inflammation, and CYP3A-dependent metabolism further limits the interpretation of this potential association. Larger prospective studies with comprehensive pharmacogenetic and functional characterization are needed to determine whether *IL-10* rs1800896 contributes to interindividual variability in tacrolimus exposure and whether it may have potential applications in individualized immunosuppressive therapy.

## 1. Introduction

Chronic kidney disease (CKD) remains a major global health burden, with kidney transplantation representing the most effective treatment option for patients with end-stage renal disease by significantly improving survival and quality of life compared with dialysis [1,2,3]. Despite advances in surgical techniques and immunosuppressive regimens, long-term graft survival is still limited by immune-mediated injury, chronic allograft dysfunction, and progressive loss of renal function. These observations indicate that variability in post-transplant outcomes cannot be explained solely by clinical or procedural factors.

Immune responses following kidney transplantation are governed by a complex interplay between innate and adaptive immunity, in which cytokines play a central regulatory role [4,5,6,7]. Cytokine-dependent signaling pathways influence T-cell activation, inflammatory amplification, and immune tolerance, thereby contributing to both acute rejection and chronic graft injury [8,9,10]. Increasing evidence also suggests that chronic inflammatory signaling affects systemic metabolism and pathways involved in drug disposition, raising the possibility that cytokine-related genetic variability may influence both graft function and immunosuppressive drug dosing.

Among cytokines involved in transplant immunology, interleukin-2 (IL-2), interleukin-6 (IL-6), and interleukin-10 (IL-10) are critical mediators that balance immune activation and suppression. IL-2 plays a fundamental role in T cell proliferation and differentiation and constitutes a primary target of calcineurin-based immunosuppressive therapy [11,12]. IL-6 functions as a multifunctional pro-inflammatory cytokine contributing to immune cell activation and tissue injury [13,14], whereas IL-10 primarily counteracts excessive inflammation and promotes immune tolerance [15,16]. Genetic variability within cytokine regulatory regions has therefore attracted attention as a potential modifier of immune responses and transplant outcomes [17,18,19].

Interleukin gene polymorphisms located in promoter regions may influence cytokine expression by altering transcriptional activity. The *IL-2* −330T>G (rs2069762) polymorphism has been associated with differences in *IL-2* gene transcription, with the G allele generally linked to higher IL-2 production than the T allele following lymphocyte activation [20,21]. Previous studies have indicated that the *IL-2* −330T>G (rs2069762) polymorphism may be associated with the risk of acute rejection in kidney transplant recipients [18].

Similarly, the *IL-6* −174G>C polymorphism (rs1800795) has been widely investigated due to its potential influence on *IL-6* expression levels and inflammatory responses. The C allele has been shown to be generally associated with lower IL-6 production than the G allele, although results are inconsistent across populations [22,23]. Variations in IL-6 production associated with this polymorphism may contribute to differences in inflammatory activity and immune-mediated tissue injury, potentially affecting transplant outcomes [19,24,25,26].

The *IL-10* −1082A>G (rs1800896) polymorphism has been shown to affect promoter activity, which is associated with higher IL-10 production for the G allele, whereas the A allele is considered a lower-producing variant [27,28,29]. Biologically, IL-10 plays a central role in immune regulation and the maintenance of peripheral tolerance, including suppression of pro-inflammatory cytokine production and modulation of antigen-presenting cell activity [15]. It is therefore plausible that inter-individual differences in IL-10 transcriptional regulation could contribute to variability in inflammatory tone and downstream organ function in chronically immunosuppressed patients. Nevertheless, the magnitude of the observed effects suggests that *IL-10* variation represents only one component within a broader immunometabolic network rather than a primary determinant of graft function [16].

Despite extensive research on cytokine gene polymorphisms in kidney transplantation, previous studies have mainly focused on clinical outcomes such as acute rejection or graft survival. Whether functional cytokine variants contribute to interindividual differences in biochemical phenotype and calcineurin inhibitor pharmacokinetics, and how these factors interact within a single integrated framework, remains largely unknown.

Therefore, the present study aimed to assess the relationships among selected cytokine gene polymorphisms (*IL-2* −330T>G, *IL-6* −174G>C, and *IL-10* −1082A>G), biochemical and hematological parameters, and calcineurin inhibitor (tacrolimus and cyclosporine) dosing in a cohort of kidney transplant recipients. We hypothesized that variability in cytokine regulatory genes—particularly within the anti-inflammatory *IL-10* axis—contributes to inter-individual differences in graft function indirectly, by modulating systemic metabolic–inflammatory status and tacrolimus exposure rather than through isolated genetic effects. We further hypothesized that these associations are genotype-dependent and influenced by age and sex, reflecting a multidimensional immunometabolic mechanism underlying long-term graft variability.

## 2. Materials and Methods

### 2.1. Patients

This cross-sectional study enrolled 394 kidney transplant recipients receiving immunosuppressive therapy, who were divided into two groups based on treatment with cyclosporine (CsA) or tacrolimus (TAC). Participants were recruited between 2014 and 2016 from the Division of Nephrology and Kidney Transplantation at the Independent Public Provincial Hospital in Szczecin and the Department of General Surgery and Transplantation at the Pomeranian Medical University in Szczecin (Poland). The study protocol was approved by the Bioethics Committee of the Poznan University of Medical Sciences, Poland (approval numbers 510/12 and 574/18), and all participants provided written informed consent prior to inclusion in the study.

Following renal transplantation, standard immunosuppressive therapy included a calcineurin inhibitor (TAC or CsA), mycophenolate mofetil, and glucocorticoids. Patients were excluded if they were underage, undergoing retransplantation, or had rapidly progressing graft failure, severe neurocognitive disorders, or an inability to communicate fluently in Polish. All consecutive adult kidney transplant recipients attending routine follow-up visits during the recruitment period who met the eligibility criteria and provided written informed consent were invited to participate.

Blood samples and clinical data were collected during routine outpatient follow-up after kidney transplantation. The study population consisted of stable kidney transplant recipients, with a median time from transplantation to sample collection of 7 years (Q1–Q3: 3.00–12.00 years). Whole-blood samples were collected immediately before the morning dose (12-h trough concentration). Peripheral blood samples were used for biochemical analyses (including renal, hepatic, and lipid parameters), measurement of drug concentrations (cyclosporine and tacrolimus), and genetic variant analysis. Fasting whole-blood levels of TAC and CsA were measured prior to drug administration. Tacrolimus and cyclosporine trough concentrations were measured using validated chemiluminescent microparticle immunoassays (CMIA) according to the manufacturer’s instructions, using the ARCHITECT Tacrolimus and ARCHITECT Cyclosporine assays, respectively (Abbott, Chicago, IL, USA) [30]. For both tacrolimus and cyclosporine, the concentration-to-dose (C/D) ratio was calculated as the trough whole-blood drug concentration (ng/mL) divided by the total daily dose (mg/day) and expressed as ng/mL per mg/day. Clinical and biochemical parameters were assessed to evaluate the risk of graft rejection.

Missing laboratory data were imputed using the closest available measurement obtained within one month before or after the study visit. Analyses were subsequently performed using a complete-case approach, with participants lacking data for any variable included in a given model excluded from that analysis. The eGFR values were calculated using the Chronic Kidney Disease Epidemiology Collaboration (CKD-EPI) equation.

### 2.2. Analysis of IL-2 rs2069762, IL-6 rs1800795, IL-10 rs1800896 Polymorphisms

Genetic analyses were performed at the Department of Cancer Immunology, Poznań University of Medical Sciences, Poland. Genomic DNA was extracted from peripheral blood leukocytes using the commercial Macherey-Nagel NucleoSpin^®^ Blood kit (Macherey-Nagel GmbH & Co., Duren, Germany), following the manufacturer’s instructions. The quality and concentration of the obtained DNA were assessed spectrophotometrically, confirming its suitability for further molecular analyses.

The *IL-2* rs2069762, *IL-6* rs1800795 and *IL-10* rs1800896 polymorphisms were analyzed using real-time PCR (LightCycler^®^ 480 system, Roche Diagnostics, Mannheim, Germany). Genotyping was conducted with the LightSNiP rs2069762, LightSNiP rs1800795, and LightSNiP *IL-10* rs1800896 assays (TIB Molbiol, Berlin, Germany), which included optimized concentrations of specific primers and probes for the target fragment. These assays were prepared according to the manufacturer’s protocol.

PCR amplification began with an initial denaturation step at 95 °C for 10 min, followed by 45 cycles consisting of denaturation at 95 °C for 10 s, annealing at 60 °C for 10 s, and extension at 72 °C for 15 s. The final step involved melting curve analysis with a temperature increase to 95 °C. Genotypes were determined based on melting curve profiles using LightCycler^®^ 480 Basic Software (version 1.5). 

To exclude the possibility of genotyping errors, genetic analysis for the *IL-6* rs1800795 and *IL-10* rs1800896 polymorphisms was performed twice, in two independent replicates. Consistent results were obtained in both analyses, confirming the accuracy of the genotype assignments and ruling out genotyping errors as the cause of the observed deviation from Hardy–Weinberg equilibrium.

### 2.3. Statistical Analysis

Statistical analysis of the study results was performed in R (version 4.3.1, http://cran.r-project.org) [31]. The distribution of continuous variables was assessed for normality using the Shapiro–Wilk test, and homogeneity of variance was checked using Levene’s test. Descriptive statistics presented arithmetic means with standard deviation (±SD) for parametric variables, and medians with lower and upper quartiles (Q1–Q3) for nonparametric variables. Parametric tests (Student’s *t*-test for two groups and ANOVA with Tukey’s post hoc test for more than two groups) and nonparametric tests (Mann–Whitney U test for two groups or Kruskal–Wallis test with Dunn’s post hoc test for more than two groups) were used to identify differences between groups. Dunn’s test was Bonferroni-corrected for multiple testing. Qualitative data were presented as numbers (N) and percentages (%), and comparisons were performed using Pearson’s χ2 test or Fisher’s exact test in small sample sizes. Chi-squared test was also used for the assessment of the Hardy–Weinberg equilibrium. Statistical significance was set at *p* < 0.05. Logistic regression was used to compare genotype frequencies between groups, and odds ratios (ORs) with 95% confidence intervals (95%CI) were calculated for five genetic models: codominant, dominant, recessive, overdominant, and log-additive. The Akaike information criterion (AIC) was used to evaluate models. Statistical analyses of genetic data were performed using the R package “SNPassoc” version 2.0-2 [32]. Linear regression was performed to confirm the associations obtained in univariate analyses and to adjust for risk factors (age, gender, time since transplantation).

To limit the risk of false-positive findings from multiple comparisons, post hoc analyses were adjusted using the Bonferroni correction. The False Discovery Rate (FDR) method was used to adjust multiple comparisons during the stratification of the research group. To determine statistical power, we used “genpwr” R package [33]. Since all of our patients had received kidney transplants, we used as a control the genotype frequency from the 1000Genomes Project’s European population to power calculation (*n* = 1006, rs2069762 MAF = 0.29, rs1800795 MAF = 0.41 and rs1800896 MAF = 0.45) [34]. The power for the test logistic model was 0.87, 0.41 and 0.98 for the dominant, recessive, and additive models, respectively, assuming a case–control rate of 0.281 for our group (*n* = 394), a level of significance of 0.05, and an OR = 1.5.

## 3. Results

### 3.1. Study Population Characteristics

**Figure 1:**
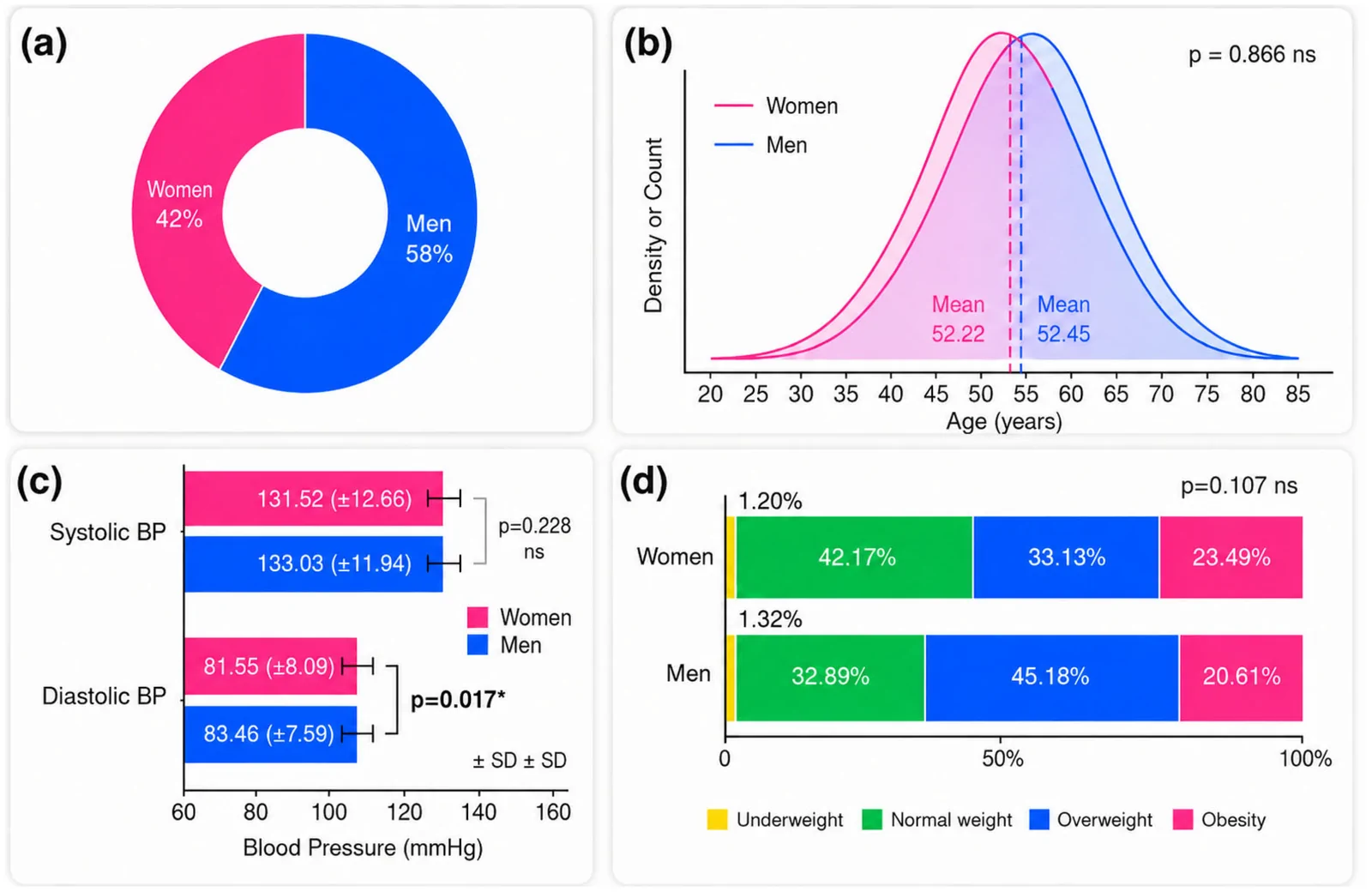
Study population characteristics. Sex distribution of the study group (**a**), with age distribution (**b**), blood pressure values (**c**), and BMI categories (**d**) presented by sex. Significant *p*-values (*p* < 0.05) are indicated by an asterisk (*).

The study included 394 adult kidney transplant recipients (166 women and 228 men) aged 19–82 years (mean ± SD: 52.36 ± 13.29 years) receiving maintenance immunosuppressive therapy. General demographic and clinical characteristics of the study population are presented in Table S1 and Figure 1.

Based on body mass index (BMI) classification, 145 patients (36.8%) had normal body weight, 158 (40.1%) were overweight, and 86 (21.8%) were obese. Underweight was observed in five individuals (1.3%). Mean BMI values did not differ significantly between women and men. Women had lower mean height and body weight than men, while systolic blood pressure was comparable between the sexes; diastolic blood pressure was slightly lower in women.

The median time since kidney transplantation was 7 years (Q1–Q3: 3–12), with no significant difference between women and men. Patients were stratified by sex, age group, and time since transplantation for subsequent analyses.

### 3.2. Analysis of Laboratory Parameters

**Figure 2:**
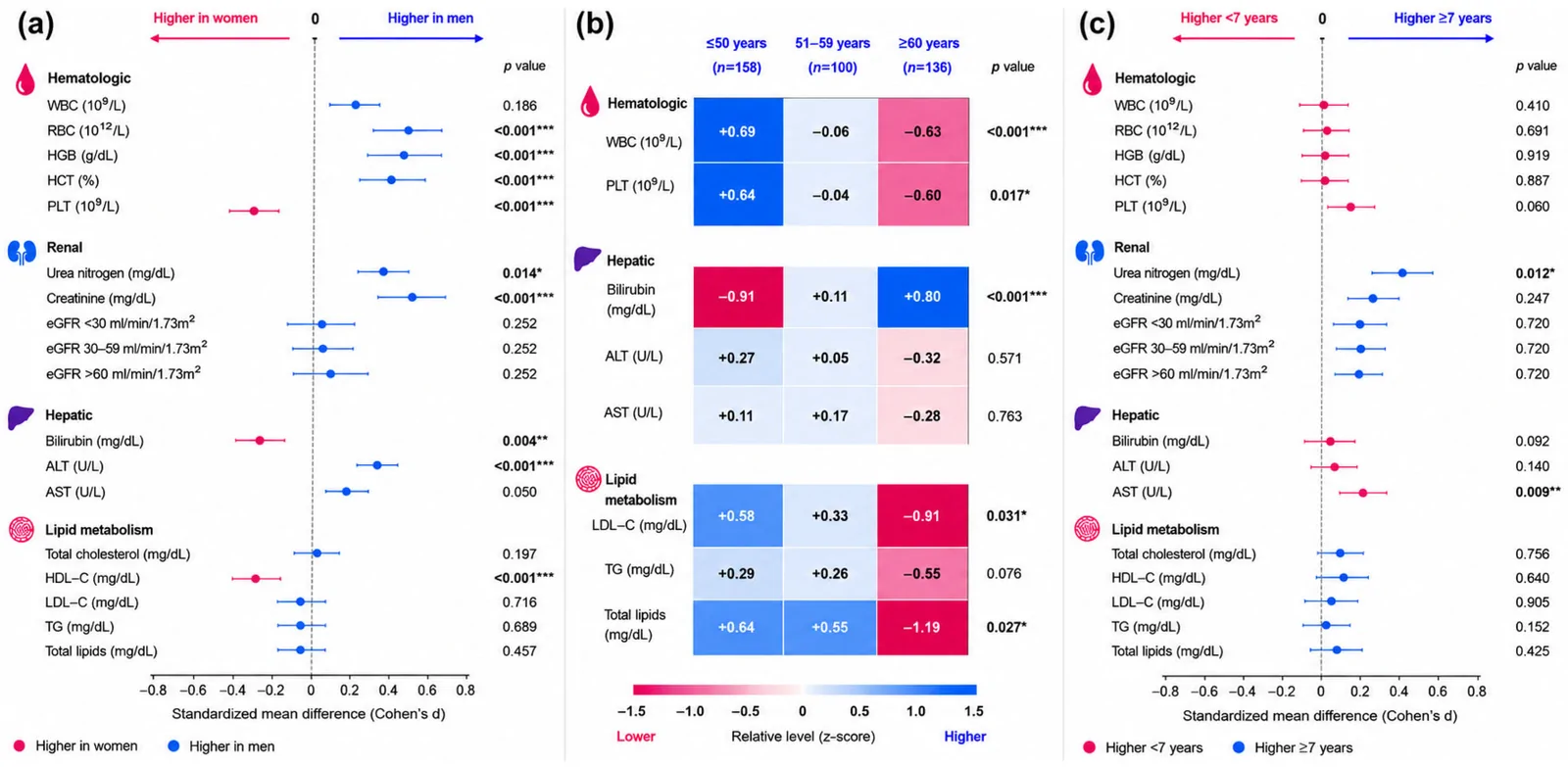
Characteristics of laboratory data of renal transplant patients treated with immunosuppressive therapy according to (**a**) gender, (**b**) age, and (**c**) time since transplantation. Panels (**a**,**c**) present standardized mean differences (Cohen’s d) with 95% CI. Panel (**b**) shows the relative levels of laboratory parameters, expressed as z-scores, within the study cohort. Blue indicates relatively higher values, whereas pink indicates relatively lower values. Significant *p*-values are indicated by asterisk: * *p* < 0.05, ** *p* < 0.01, and *** *p* < 0.001.

Laboratory parameters were analyzed according to sex, age group, and time since transplantation summarized in Figure 2 and Tables S2–S4.

As expected, analysis of mean parameters revealed statistically significant differences between men and women for many of them. Median total white blood cell (WBC) counts were 7.47 [Q1–Q3: 6.04–9.46] for women and 7.79 [Q1–Q3: 6.33–9.56] for men (*p* = 0.186). Women had statistically significantly lower mean values of red blood cells (4.15 ± 0.57 vs. 4.50 ± 0.61, *p* < 0.001), hemoglobin (12.41 ± 1.62 g/dL vs. 13.55 ± 1.78 g/dL, *p* < 0.001), and hematocrit (36.88 ± 4.57% vs. 39.92 ± 4.96%, *p* < 0.001) compared to men. In men, statistically significant lower median values for platelets were observed: 195.00 [Q1–Q3: 159.00–242.50] compared to 217.00 [Q1–Q3: 180.00–268.50] in women (*p* < 0.001). Comparison of biochemical parameters between women and men in the study group revealed lower, statistically significant medians for bilirubin (*p* = 0.004), urea nitrogen (*p* = 0.014), and creatinine (*p* < 0.001) in women. Alanine transaminases and aspartate transaminases also had lower medians in this group (ALT: 14.00 U/L [Q1–Q3: 10.00–19.00] vs. 18.00 U/L [Q1–Q3: 14.00–27.00], *p* < 0.001; AST: 17.00 U/L [Q1–Q3: 14.00–22.00] vs. 19.00 U/L [Q1–Q3: 16.00–24.00], *p* = 0.050). When comparing blood electrolytes, it was observed that patients from the study group had a statistically significantly lower median value for potassium (*p* = 0.024), and lower values for sodium, but without statistical significance (141.00 mmol/L [Q1–Q3: 139.00–142.70] vs. 141.00 mmol/L [Q1–Q3: 140.00–144.00], *p* = 0.067). Analyzing the lipid profile, lower values of high-density lipoproteins (HDLs) were noted for men compared to the women group (58.15 mg/dL [Q1–Q3: 42.35–68.15] vs. 65.60 mg/dL [Q1–Q3: 54.50–76.60], *p* < 0.001).

Age-stratified analyses revealed significant changes in selected biochemical and hematological parameters. A gradual increase in median bilirubin concentration was observed with advancing age. In parallel, lipid parameters decreased across age groups, including total lipid levels and low-density lipoprotein (LDL) cholesterol (LDL-C), with the lowest values recorded in patients aged ≥60 years. These trends were consistent across the age categories analyzed. In assessed hematological parameters, increasing age was associated with lower total leukocyte and platelet counts. Moreover, analysis of renal function parameters by age revealed no statistically significant differences between the analyzed age groups. Creatinine concentration (*p* = 0.183), urea nitrogen concentration (*p* = 0.078), and the distribution of eGFR categories (*p* = 0.090) were comparable across age groups.

Time since transplantation did not significantly affect hematological parameters. However, patients transplanted for ≥7 years demonstrated significantly higher urea nitrogen and aspartate aminotransferase (AST) levels than those transplanted for <7 years, indicating time-dependent differences in selected biochemical indices.

### 3.3. Distribution of Cytokine Gene Polymorphisms According to Immunosuppressive Regimen

Patients were stratified according to maintenance immunosuppressive regimen: 184 (46.7%) received cyclosporine (CsA), and 210 (53.3%) received tacrolimus (TAC). The distribution of sex and age did not differ significantly between treatment groups.

Hardy–Weinberg equilibrium (HWE) was assessed separately for each analyzed cytokine polymorphism. Genotype frequencies for the *IL-2* *rs2069762* polymorphism were consistent with HWE (χ^2^ = 0.124, *p* = 0.939). In contrast, significant deviations from HWE were observed for *IL-6* rs1800795 (χ^2^ = 7.998, *p* = 0.018) and *IL-10* rs1800896 (χ^2^ = 6.999, *p* = 0.030). The observed deviations from HWE may be attributable to the analysis being conducted within the study group, where disease-associated genetic factors could influence the distribution of genotypes.

**Figure 3:**
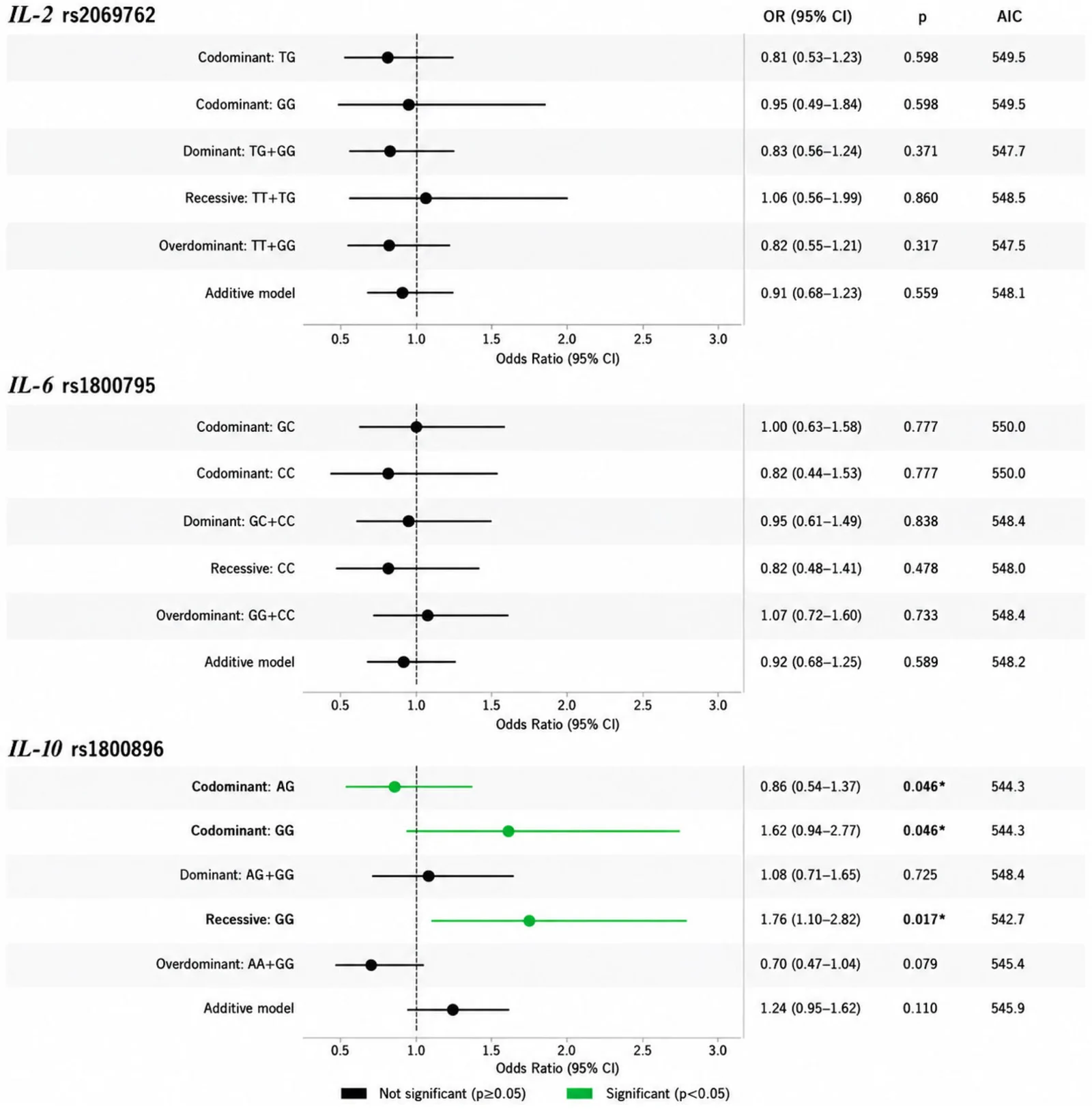
Forest plots presenting odds ratios (ORs) and 95% confidence intervals for comparisons of *IL-2*, *IL-6*, and *IL-10* genotype distributions between CsA and TAC-treated kidney transplant recipients under codominant, dominant, recessive, overdominant, and additive genetic models. Significant *p*-values (*p* < 0.05) are indicated by an asterisk (*).

Analysis of the *IL-2* rs2069762 polymorphism showed comparable genotype frequencies in CsA- and TAC-treated patients. No statistically significant differences were observed between treatment groups across codominant, dominant, recessive, overdominant, or additive genetic models (Table S5, Figure 3).

Similarly, the genotype distribution of the *IL-6* rs1800795 polymorphism did not differ significantly between the CsA and TAC groups across any of the evaluated genetic models (Table S6, Figure 3).

In contrast, genotype distribution for the *IL-10* rs1800896 polymorphism differed between immunosuppressive regimens. The homozygous GG genotype was more frequently observed among TAC-treated patients compared with CsA-treated patients, whereas genotypes carrying the A allele (AA and AG) were more prevalent in the CsA group. Logistic regression analysis indicated that the recessive model provided the best fit for this polymorphism (Table S7, Figure 3).

Overall, among the analyzed cytokine polymorphisms, only *IL-10* rs1800896 showed a genotype distribution that varied by immunosuppressive regimen, whereas *IL-2* and *IL-6* polymorphisms showed no evidence of treatment-dependent differences.

### 3.4. Association Between Cytokine Gene Polymorphisms and Clinical and Laboratory Parameters

Associations between cytokine gene polymorphisms and selected clinical and laboratory parameters were assessed in the entire study cohort. Analyses included genotype- and allele-based comparisons for *IL-2* rs2069762, *IL-6* rs1800795, and *IL-10* rs1800896.

No statistically significant associations were observed between *IL-2* rs2069762 genotypes or alleles and age, body mass index, blood pressure, or any hematological or biochemical parameters (Table 1 and Table 2).

Similarly, *IL-6* rs1800795 genotypes and alleles were not associated with clinical variables, renal function indices, liver enzymes, or lipid profile, except for a modest genotype-dependent difference in bilirubin concentration. Specifically, median bilirubin levels differed between CC and GC genotypes, whereas no significant differences were observed at the allele level (Table 3).

A Kruskal–Wallis H test was conducted to examine differences in biochemical parameters across *IL-10* genotypes: AA (*n* = 125), AG (*n* = 170), and GG (*n* = 90). Significant differences were observed in renal function markers, including urea nitrogen and eGFR, across *IL-10* genotypes (Table 4). Median urea nitrogen was 22.25 mg/dL [IQR: 17.90–34.85] for AA, 27.05 mg/dL [IQR: 20.10–38.00] for AG, and 23.15 mg/dL [IQR: 17.90–30.00] for GG. The Kruskal–Wallis test indicated a statistically significant difference, H(2) = 8.921, *p* = 0.012, eta2 = 0.023 (small effect size), 95%CI: 0.005–0.065. Post hoc pairwise comparisons using Dunn’s test with Bonferroni correction revealed significant differences between genotypes AA vs. GA (z = 2.53, *p* = 0.011, padj = 0.034), and GA vs. GG (z = −2.45, *p* = 0.014, padj. = 0.042). The difference between homozygous genotypes was not statistically significant for AA vs. GG (z = −0.092, *p* = 0.927, padj = 1.000). A chi-square test was conducted to examine the relationship between eGFR and IL10 genotypes. The relation between these variables was significant, χ^2^(4, *n* = 394) = 14.659, *p* = 0.005, Cramer’s V = 0.136 indicating a weak association.

Median alanine aminotransferase (ALT) and aspartate aminotransferase (AST) values were highest in carriers of the GG genotype, with statistically significant differences observed for ALT and borderline significance for AST. The medians for ALT were: 16.00 (U/L) [IQR: 12.00–20.00], 17.00 (U/L) [IQR: 12.00–26.00] and 18.00 (U/L) [IQR: 13.00–26.00] for AA, AG and GG, respectively. The Kruskal–Wallis test indicated a statistically significant difference, H(2) = 6.1527, *p* = 0.046, eta2 = 0.016 (small effect size), 95%CI: 0.003–0.051. Post hoc Dunn’s test with Bonferroni correction revealed significant differences between genotypes AA vs. GG (z = 2.47, *p* = 0.013, padj = 0.040). No significant genotype or allele-dependent associations were identified for hematological parameters, electrolyte concentrations, or lipid profile in relation to *IL-10* rs1800896.

A simple linear regression was conducted to predict urea nitrogen, eGFR and ALT from IL10 genotypes (Table 5). The models did not significantly predict ALT (F(2, 391) = 0.620, *p* = 0.539, R^2^ = 0.003) and urea nitrogen (F(2, 391) = 1.058, *p* = 0.348, R^2^ = 0.005). Based on the results, a statistically significant association was observed between homozygotes GG and eGFR (B = 7.595, SE = 3.608, β = 0.12, t = 2.105, *p* = 0.036, 95% CI: 0.50, 14.69). However, the association strength was weak (adjusted R square = 0.006) and no longer observed after the age, sex, and time since transplantation adjustments (B = 6.800, SE = 3.608, β = 0.11, t = 1.884, *p* = 0.060, 95% CI: −0.29, 13.89). Overall, among the analyzed cytokine gene variants, *IL-10* rs1800896 was the only polymorphism associated with genotype-dependent differences in renal and hepatic biochemical parameters in univariate analyses. However, these associations did not remain significant after adjustment for age, sex, and time since transplantation. In contrast, *IL-2* and *IL-6* polymorphisms showed no clinically relevant associations with the laboratory outcomes analyzed.

### 3.5. Association of Cytokine Gene Polymorphisms with Calcineurin Inhibitor Dosing

To evaluate whether cytokine gene polymorphisms were associated with variability in immunosuppressive therapy, analyses were performed for drug dose, blood concentration, and concentration-to-dose (C/D) ratio in patients receiving CsA or TAC.

For the *IL-2* rs2069762 polymorphism, no statistically significant differences were observed between genotypes regarding CsA or TAC blood concentrations, daily doses, or C/D ratios (Table 6). Although a gradual increase in the tacrolimus C/D ratio across *IL-2* genotypes was observed, this trend did not reach statistical significance. Similarly, *IL-6* rs1800795 genotypes were not associated with significant differences in immunosuppressant concentrations, dosing, or C/D ratios for either CsA or TAC (Table 6).

In contrast, analysis of the *IL-10* rs1800896 polymorphism revealed genotype-dependent differences in tacrolimus C/D ratio. While tacrolimus blood concentrations and daily doses did not differ significantly across *IL-10* genotypes, the tacrolimus C/D ratio varied significantly, with the lowest median values observed in carriers of the AG genotype compared with AA and GG genotypes (Table 6). No significant associations between *IL-10* genotypes and CsA dosing were identified.

Sex-stratified analyses demonstrated limited and inconsistent genotype-dependent effects. In men receiving CsA, *IL-2* rs2069762 genotypes were associated with differences in cyclosporine blood concentrations, whereas no corresponding differences in daily dose or C/D ratio were observed (Table 7). For *IL-6* rs1800795, no significant sex-specific associations with immunosuppressant dosing were detected. In women treated with tacrolimus, *IL-10* rs1800896 genotypes were associated with significant differences in tacrolimus blood concentrations. However, these differences were not accompanied by consistent changes in daily dose or C/D ratio. Nevertheless, this association was no longer seen following FDR correction for multiple comparisons (Table 7).

The relationship between *IL-10* rs1800896 genotypes and blood level, dosages and C/D ratio of tacrolimus and cyclosporine A was evaluated using linear regression analysis. Rs1800896 genotypes did not predict CsA level (F(2, 181) = 0.832, *p* = 0.437, R^2^ = 0.009), dose (F(2, 181) = 0.381, *p* = 0.684, R^2^ = 0.004) and C/D ratio (F(2, 181) = 0.246, *p* = 0.782, R^2^ = 0.003). Additionally, *IL-10* genotypes did not statistically significantly influence the immunosuppressant level in blood (F(2, 207) = 0.476, *p* = 0.622, R^2^ = 0.005) and dose (F(2, 207) = 1.261, *p* = 0.286, R^2^ = 0.012) among patients receiving TAC. The model with the C/D ratio was nearest to statistical significance (F(2, 207) = 3.048, *p* = 0.05, R^2^ = 0.029). For the heterozygous GA genotype, values of B = −0.462, SE = 0.256, β = −0.15, t = −1.807, *p* = 0.072, 95% CI: −0.97, 0.04 were obtained, and after age, sex, and time since transplantation adjustments, these were B = −0.450, SE = 0.254, β = −0.14, t = −1.772, *p* = 0.078, 95% CI: −0.95, 0.05 (Table 8).

## 4. Discussion

Kidney transplantation is the optimal treatment for end-stage renal disease; however, long-term graft survival remains limited by mechanisms extending beyond acute alloimmune rejection, including chronic low-grade inflammation, metabolic dysregulation, and progressive graft dysfunction. Increasing evidence indicates that these processes are governed by complex polygenic and systemic interactions rather than isolated genetic variants or single immune pathways [6,35,36,37,38]. In this context, the present study provides an integrated analysis of cytokine gene polymorphisms, biochemical phenotype, and calcineurin inhibitor dosing in a large cohort of kidney transplant recipients.

Previous studies from our group investigated the contribution of selected immunoregulatory gene polymorphisms to calcineurin inhibitor therapy in kidney transplant recipients. This study focused mainly on tacrolimus dose requirements and selected clinical and biochemical parameters, demonstrating a potential association between *IL-10* genotype and tacrolimus dose, while no significant relationships with renal biochemical parameters or rejection risk were identified [39]. In another study, we investigated the influence of *CTLA4* rs231775 and *TGFB1* rs1800468 polymorphisms on calcineurin inhibitor therapy in 392 kidney transplant recipients [40]. Although the present cohort partially overlaps with populations described in these previous studies, the objectives and genetic analyses are distinct. The previous study focused on variants involved in T-cell activation and TGF-β signaling, whereas the current study evaluates cytokine polymorphisms and their potential association with calcineurin inhibitor pharmacokinetic variability. The present study extends these previous observations by evaluating *IL-10* rs1800896 together with additional cytokine polymorphisms, including *IL-2* rs2069762 and *IL-6* rs1800795, and by assessing their associations with both tacrolimus and cyclosporine exposure using concentration-to-dose (C/D) ratio analyses and multivariable statistical models.

The main finding of this study is that, among the cytokine gene polymorphisms analyzed, *IL-10* −1082A>G (rs1800896) showed a potential association with tacrolimus exposure, as reflected by differences in the concentration-to-dose ratio (C/D), whereas *IL-2* (rs2069762) and *IL-6* (rs1800795) polymorphisms did not demonstrate statistically significant associations with calcineurin inhibitor exposure or clinically relevant biochemical parameters. Although some associations between *IL-10* rs1800896 and selected biochemical parameters were observed in univariate analyses, these results were not confirmed in multivariate models. Therefore, the observed associations should be interpreted with caution and considered exploratory.

The cytokine polymorphisms analyzed were not associated with graft rejection, graft survival, patient survival, or clinically relevant markers of kidney transplant function. These results suggest that the cytokine genetic variants studied, including *IL-10* rs1800896, are unlikely to have a significant, detectable impact on long-term transplant outcomes in the current cohort. However, the potential association between *IL-10* genetic variation and tacrolimus exposure may require further investigation, especially given the significant interindividual variability in tacrolimus pharmacokinetics.

Regarding tacrolimus pharmacokinetics, regression analysis revealed a borderline correlation at the overall model level, whereas the genotype-specific association for the heterozygous GA genotype did not reach statistical significance after adjustment for age, sex, and time since transplantation. Therefore, the present results do not provide sufficient evidence to conclude that *IL-10* rs1800896 is independently associated with tacrolimus exposure. Nevertheless, the observed pattern is consistent with previous reports suggesting a possible association between cytokine gene variation and calcineurin inhibitor pharmacokinetics. Seyhun et al. described associations between cytokine gene polymorphisms, including *IL-10* variants, and blood calcineurin inhibitor concentrations in renal transplant recipients, while Stefanović et al. demonstrated that *IL-6* and *IL-10* genotypes can influence tacrolimus requirements using pharmacogenomic modeling methods [41,42]. These findings therefore provide a basis for further investigation of *IL-10* rs1800896 in relation to tacrolimus exposure but should not be interpreted as definitive proof of pharmacokinetic effects.

Further studies in larger and independent cohorts, including comprehensive pharmacogenetic characterization and relevant clinical and functional variables, are needed to determine whether the observed association represents a reproducible biological effect. In particular, the inclusion of *CYP3A5* genotyping and functional assessment of cytokine activity and CYP3A-dependent metabolism may help clarify whether *IL-10* related genetic variability contributes significantly to interindividual differences in tacrolimus pharmacokinetics.

The biological mechanism underlying the observed relationship remains uncertain. Previous experimental and clinical studies have suggested that inflammatory signaling, cytokine regulation, and immunometabolic status may modulate hepatic drug-metabolizing capacity, including CYP3A-dependent pathways involved in tacrolimus biotransformation [43,44,45,46,47]. In this context, the present findings may be compatible with the hypothesis that genetic variation affecting IL-10 activity could contribute indirectly to interindividual differences in tacrolimus pharmacokinetics through modulation of inflammatory signaling and drug-metabolizing pathways. However, this study was not designed to investigate these underlying biological mechanisms, and no causal relationship can therefore be inferred. The lack of significant associations for the *IL-2* and *IL-6* promoter polymorphisms observed in the present study is consistent with previous meta-analytic evidence indicating limited or absent effects of the *IL-2* −330T>G variant on renal transplant outcomes [45]. More broadly, prior meta-analyses and cohort studies have reported heterogeneous and largely inconsistent associations between individual pro-inflammatory cytokine polymorphisms and graft outcomes [35]. Although *IL-6*-mediated inflammation has been implicated in graft fibrosis and rejection in experimental and clinical settings [13,14,24,25,26], the present data suggest that, under long-term immunosuppression, variation within *IL-2* and *IL-6* regulatory regions may be effectively buffered by pharmacological inhibition and compensatory immune mechanisms.

Beyond the analysis of calcineurin inhibitor exposure, we also evaluated the potential clinical relevance of the investigated cytokine gene polymorphisms (*IL-2* −330T>G, *IL-6* −174G>C, and IL-10 −1082A>G). Their associations with biochemical markers of kidney graft function, including estimated glomerular filtration rate (eGFR), serum creatinine, urea nitrogen, and electrolyte levels, as well as with clinical outcomes such as graft rejection, graft survival, and patient survival, were assessed. No significant associations were observed between the analyzed polymorphisms and increased risk of graft rejection, impaired graft function, or reduced graft or patient survival. These findings suggest that, despite their potential involvement in inflammatory regulation, the studied cytokine variants do not appear to be major determinants of long-term transplant outcomes in this cohort.

Age and sex emerged as important modulators of the biochemical phenotype but did not account for the genotype-specific effects observed for *IL-10*. Age-related increases in bilirubin and reductions in lipid parameters, as well as sex-related differences in hematological and metabolic markers, are consistent with established physiological patterns in transplant cohorts and reflect systemic background variability rather than primary genetic effects.

Although oxidative stress markers were not directly assessed in this study, the observed associations are biologically compatible with a model in which *IL-10*-dependent regulation of chronic low-grade inflammation may indirectly influence oxidative balance and metabolic stress. Chronic inflammatory signaling is closely linked to the generation of reactive oxygen species, mitochondrial dysfunction, and alterations in hepatocellular metabolism, which, in turn, may affect drug disposition and organ function. In this regard, regulatory cytokines such as *IL-10* may function as system-level hubs that integrate inflammatory, metabolic, and oxidative processes, rather than acting as isolated immune mediators. This systems-based perspective is consistent with conceptual frameworks that describe chronic inflammation and oxidative stress as interconnected drivers of disease chronicity [48].

Several limitations should be considered when interpreting these findings. First, the cross-sectional design precludes conclusions regarding causality. Second, although the regression model for tacrolimus C/D ratio showed a borderline association at the overall model level, the genotype-specific association for *IL-10* rs1800896 did not reach statistical significance after adjustment for age, sex, and time since transplantation. Therefore, the present findings should be interpreted as exploratory and do not provide sufficient evidence for an independent effect of *IL-10* rs1800896 on tacrolimus pharmacokinetics. Importantly, the CYP3A5 genotype, the main pharmacogenetic determinant of tacrolimus metabolism, was not available in the current cohort. Therefore, residual confounding due to CYP3A5 variability or other established and potentially relevant pharmacogenetic factors cannot be excluded.

Additional limitations include the lack of information on several important determinants of tacrolimus exposure, such as liver function, tacrolimus formulation, dosing interval, concomitant medications, corticosteroid therapy, and medication adherence. Finally, multiple genetic inheritance models were evaluated for each polymorphism. Although this approach was intended to comprehensively explore potential genotype–phenotype relationships, it increases the risk of type I error. Therefore, the observed associations should be regarded as exploratory and require confirmation in larger, prospective studies with comprehensive clinical and pharmacogenetic characterization. Moreover, the deviations from Hardy–Weinberg equilibrium (HWE) observed for the *IL-6* and *IL-10* polymorphisms should be interpreted with caution. Genotyping errors are unlikely to explain these findings, as both polymorphisms were genotyped twice in two independent analyses, yielding complete concordance between the results. The observed HWE deviations are more likely related to the characteristics of the study population, which consisted exclusively of renal transplant recipients, in whom genotype distributions may differ from those of the general population because of disease-related selection or other factors associated with transplantation. Nevertheless, deviation from HWE remains a limitation of the study. Therefore, the association between *IL-10* rs1800896 and the tacrolimus concentration-to-dose (C/D) ratio should be considered exploratory and requires confirmation in larger, independent cohorts with genotype distributions consistent with Hardy–Weinberg equilibrium.

## 5. Conclusions

In this cohort of kidney transplant recipients, the *IL-10* rs1800896 genotype demonstrated a possible association with tacrolimus exposure, assessed by the C/D ratio, whereas no statistically significant associations were observed for the analyzed *IL-2* rs2069762 and *IL-6* rs1800795 variants. The potential association between *IL-10* rs1800896 and the tacrolimus C/D ratio did not reach statistical significance in the adjusted analysis. No significant associations were observed between the cytokine polymorphisms studied and graft rejection, graft survival, patient survival, or clinically relevant markers of kidney transplant function.

These results suggest a potential contribution of *IL-10* rs1800896 to interindividual variability in tacrolimus exposure. However, this observation should be considered preliminary and interpreted with caution. Further prospective studies with larger cohorts and comprehensive pharmacogenetic characterization, including *CYP3A5* genotyping and functional assessment of cytokine activity and CYP3A-dependent metabolism, are needed to determine whether *IL-10* rs1800896 significantly contributes to the pharmacokinetic variability of tacrolimus and whether it may have potential use in individualized immunosuppressive therapy.

## Figures and Tables

**Table 1:** Associations of *IL-2* rs2069762, *IL-6* rs1800795, and *IL-10* rs1800896 genotypes with clinical data of patients after kidney transplantation.

*IL-2* rs2069762	TT (*n* = 170)	TG (*n* = 180)	GG (*n* = 44)	*p*
Age, years, mean ± SD	53.04 ± 12.62	51.77 ± 13.10	52.11 ± 16.44	0.665
Age category, *n* (%):				0.504
≤50 years	63 (37.06)	74 (41.11)	21 (47.73)	
51–59 years	45 (26.47)	48 (26.67)	7 (15.91)	
≥60 years	62 (36.47)	58 (32.22)	16 (36.36)	
Sex, *n* (%)				0.602
Female	76 (44.71)	71 (39.44)	19 (43.18)	
Male	94 (55.29)	109 (60.56)	25 (56.82)	
BMI, kg/m^2^, mean ± SD	26.59 ± 4.78	26.90 ± 4.73	27.21 ± 4.98	0.694
BMI category, *n* (%):				0.174
Underweight (<18.5)	1 (0.59)	4 (2.22)	0 (0.00)	
Normal weight (18.5–24.99)	73 (42.94)	58 (32.22)	14 (31.82)	
Overweight (25.0–29.99)	57 (33.53)	81 (45.00)	20 (45.45)	
Obesity (≥30)	39 (22.94)	37 (20.56)	10 (22.73)	
Systolic BP, mmHg	133.06 ± 12.21	131.96 ± 12.72	131.59 ± 10.44	0.633
Diastolic BP, mmHg	83.21 ± 7.59	82.23 ± 8.11	82.27 ± 7.81	0.484
Time since transplantation, *n* (%)				0.260
<7 years	78 (45.88)	86 (47.78)	15 (34.09)	
≥7 years	92 (54.12)	94 (52.22)	29 (65.91)	
***IL-6*** **rs1800795**	**GG (*n* = 109)**	**GC (*n* = 222)**	**CC (*n* = 63)**	***p***
Age, years, mean ± SD	52.83 ± 13.02	51.61 ± 13.26	54.16 ± 13.84	0.368
Age category, *n* (%):				0.444
≤50 years	43 (39.45)	93 (41.89)	22 (34.92)	
51–59 years	31 (28.44)	56 (25.23)	13 (20.63)	
≥60 years	35 (32.11)	73 (32.88)	28 (44.44)	
Sex, *n* (%)				0.655
Female	43 (39.45)	98 (44.14)	25 (39.68)	
Male	66 (60.55)	124 (55.86)	38 (60.32)	
BMI, kg/m^2^, mean ± SD	26.31 ± 4.47	26.95 ± 4.73	27.16 ± 5.39	0.424
BMI category, *n* (%):				0.175
Underweight (<18.5)	1 (0.92)	3 (1.35)	1 (1.59)	
Normal weight (18.5–24.99)	48 (44.04)	72 (32.43)	25 (39.68)	
Overweight (25.0–29.99)	45 (41.28)	93 (41.89)	20 (31.75)	
Obesity (≥30)	15 (13.76)	54 (24.32)	17 (26.98)	
Systolic blood pressure, mmHg	132.18 ± 12.40	132.32 ± 12.34	133.02 ± 11.86	0.903
Diastolic blood pressure, mmHg	82.22 ± 8.04	82.55 ± 7.19	83.81 ± 9.58	0.421
Time since transplantation, *n* (%)				0.849
<7 years	47 (43.12)	103 (46.40)	29 (46.03)	
≥7 years	62 (56.88)	119 (53.60)	34 (53.97)	
***IL-10*** **rs1800896**	**AA (*n* = 125)**	**AG (*n* = 170)**	**GG (*n* = 99)**	***p***
Age, years, mean ± SD	51.97 ± 13.14	53.12 ± 13.26	51.53 ± 13.58	0.590
Age category, *n* (%):				0.758
≤50 years	54 (43.20)	67 (39.41)	37 (37.37)	
51–59 years	27 (21.60)	44 (25.88)	29 (29.29)	
≥60 years	44 (35.20)	59 (34.71)	33 (33.33)	
Sex, *n* (%)				0.150
Female	61 (48.80)	69 (40.59)	36 (36.36)	
Male	64 (51.20)	101 (59.41)	63 (63.64)	
BMI, kg/m^2^, mean ± SD	26.56 ± 4.62	27.05 ± 4.75	26.68 ± 5.03	0.665
BMI category, *n* (%):				0.102
Underweight (<18.5)	0 (0.00)	1 (0.59)	4 (4.04)	
Normal weight (18.5–24.99)	52 (41.60)	59 (34.71)	34 (34.34)	
Overweight (25.0–29.99)	49 (39.20)	68 (40.00)	41 (41.41)	
Obesity (≥30)	24 (19.20)	42 (24.71)	20 (20.20	
Systolic blood pressure, mmHg	132.48 ± 10.95	133.20 ± 13.71	130.91 ± 11.10	0.337
Diastolic blood pressure, mmHg	82.92 ± 8.14	82.31 ± 8.07	82.93 ± 7.11	0.744
Time since transplantation, *n* (%)				0.670
<7 years	53 (42.40)	81 (47.65)	45 (45.45)	
≥7 years	72 (57.60)	89 (52.35)	54 (54.55)	

Abbreviations: AA—homozygous A/A genotype; AG—heterozygous A/G genotype; BMI—body mass index; BP—blood pressure; CC—homozygous C/C genotype; GC—heterozygous G/C genotype; GG—homozygous G/G genotype; IL—interleukin; SD—standard deviation; TG—heterozygous T/G genotype; TT—homozygous T/T genotype.

**Table 2:** Influence of *IL-2* rs2069762 polymorphism on hematological and biochemical parameters in the entire study group.

Parameter	TT (*n* = 170)	TG (*n* = 180)	GG (*n* = 44)	*p*
Hematological parameters				
WBC (×10^9^/L)	7.42 [6.08–8.89]	7.76 [6.30–9.98]	8.07 [6.63–9.53]	0.133
RBC (×10^12^/L)	4.37 ± 0.63	4.35 ± 0.60	4.35 ± 0.62	0.943
HGB (g/dL)	13.04 ± 1.76	13.10 ± 1.84	13.08 ± 1.85	0.956
HCT (%)	38.63 ± 4.93	38.66 ± 5.12	38.69 ± 5.04	0.997
PLT (×10^9^/L)	202.00 [159.50–253.00]	204.00 [169.50–250.50]	208.50 [179.50–266.50]	0.641
Electrolytes				
Na (mmol/L)	141.00 [139.00–144.00]	141.00 [139.00–143.00]	141.00 [140.00–143.00]	0.885
K (mmol/L)	4.11 [3.75–4.46]	4.14 [3.91–4.35]	4.15 [3.92–4.42]	0.759
Kidney function				
Urea nitrogen (mg/dL)	25.60 [19.00–35.60]	25.80 [21.30–34.85]	23.40 [18.00–32.85]	0.296
Creatinine (mg/dL)	1.46 [1.20–1.91]	1.40 [1.19–1.83]	1.44 [1.17–1.80]	0.549
eGFR mL/min/1.73 m^2^	48.68 [35.77;59.71]	52.64 [40.49;72.75]	48.44 [36.50;72.70]	0.183
eGFR category, *n* (%):				0.630
<30 mL/min/1.73 m^2^	28 (16.47)	22 (12.22%)	6 (13.64%)	
30–59 mL/min/1.73 m^2^	101 (59.41)	103 (57.22%)	25 (56.82%)	
≥60 mL/min/1.73 m^2^	41 (24.12)	55 (30.56%)	13 (29.55%)	
Liver function				
Bilirubin (mg/dL)	0.49 [0.36–0.64]	0.53 [0.38–0.61]	0.50 [0.38–0.68]	0.737
ALT (U/L)	17.00 [12.00–25.00]	17.00 [13.00–21.00]	17.00 [12.00–24.00]	0.979
AST (U/L)	19.00 [15.00–23.00]	18.00 [16.00–23.00]	18.00 [14.50–22.00]	0.736
Lipid profile				
Total cholesterol (mg/dL)	185.00 [168.00–213.00]	182.50 [156.50–203.50]	185.00 [160.50–213.00]	0.555
HDL-C (mg/dL)	63.20 [52.60–75.90]	56.85 [41.45–73.60]	58.20 [45.15–73.50]	0.128
LDL-C (mg/dL)	92.00 [76.00–115.00]	101.00 [70.50–119.00]	94.00 [73.50–119.00]	0.931
TG (mg/dL)	125.00 [96.00–179.00]	122.50 [100.50–159.50]	137.00 [97.00–180.00]	0.633
Total lipids (mg/dL)	641.00 [547.00–715.00]	601.00 [565.00–685.00]	620.50 [551.50–734.00]	0.644

Data are presented as mean ± SD or median [Q1–Q3], or *n* (%) as appropriate, p—Kruskal–Wallis test and Pearson’s Chi-squared test for eGFR categories. Abbreviations: ALT—alanine aminotransferase; AST—aspartate aminotransferase; eGFR—estimated glomerular filtration rate; HCT—hematocrit; HDL-C—high-density lipoprotein cholesterol; HGB—hemoglobin; LDL-C—low-density lipoprotein cholesterol; PLT—platelet count; RBC—red blood cell count; SD—standard deviation; TG—triglycerides; WBC—white blood cell count.

**Table 3:** Influence of *IL-6* rs1800795 polymorphism on hematological and biochemical parameters in the entire study group.

Parameter	GG (*n* = 109)	GC (*n* = 222)	CC (*n* = 63)	*p*
Hematological parameters				
WBC (×10^9^/L)	7.62 [6.25–9.30]	7.71 [6.25–9.83]	7.38 [6.03–9.48]	0.512
RBC (×10^12^/L)	4.28 ± 0.58	4.39 ± 0.60	4.36 ± 0.71	0.313
HGB (g/dL)	13.03 ± 1.71	13.09 ± 1.79	13.05 ± 2.03	0.956
HCT (%)	38.35 ± 4.83	38.82 ± 4.96	38.56 ± 5.59	0.726
PLT (×10^9^/L)	192.00 [160.00–247.50]	209.00 [167.50–253.50]	204.00 [164.00–253.00]	0.377
Electrolytes				
Na (mmol/L)	141.00 [139.00–143.00]	141.00 [139.00–143.00]	142.00 [140.00–144.00]	0.078
K (mmol/L)	4.05 [3.78–4.41]	4.16 [3.87–4.42]	4.24 [3.90–4.48]	0.197
Kidney function				
Urea nitrogen (mg/dL)	25.60 [19.55–35.10]	24.50 [18.80–34.10]	25.20 [17.90–38.00]	0.794
Creatinine (mg/dL)	1.45 [1.17–1.90]	1.44 [1.18–1.84]	1.44 [1.19–1.96]	0.966
eGFR mL/min/1.73 m^2^	50.86 [38.53;71.20]	49.79 [37.93;62.30]	51.82 [35.20;68.40]	0.915
eGFR category, *n* (%):				0.461
<30 mL/min/1.73 m^2^	20 (18.35)	27 (12.16)	9 (14.29)	
30–59 mL/min/1.73 m^2^	57 (52.29)	137 (61.71)	35 (55.56)	
≥60 mL/min/1.73 m^2^	32 (29.36)	58 (26.13)	19 (30.16)	
Liver function				
Bilirubin (mg/dL)	0.52 [0.37–0.72]	0.46 [0.35–0.61]	0.54 [0.42–0.72]	0.014
ALT (U/L)	18.00 [12.00–24.00]	16.00 [12.00–24.00]	17.00 [12.00–24.00]	0.925
AST (U/L)	19.00 [15.00–24.00]	18.00 [15.00–22.00]	18.00 [14.00–22.00]	0.356
Lipid profile				
Total cholesterol (mg/dL)	187.50 [164.00–214.00]	185.00 [162.00–211.00]	177.00 [160.00–208.00]	0.555
HDL-C (mg/dL)	62.70 [52.45–73.70]	60.60 [46.60–75.65]	57.20 [39.70–71.60]	0.218
LDL-C (mg/dL)	99.00 [74.50–115.00]	92.00 [75.00–118.00]	95.00 [73.00–123.00]	0.904
TG (mg/dL)	135.00 [98.50–184.00]	125.00 [95.00–171.00]	139.00 [102.00–190.00]	0.585
Total lipids (mg/dL)	642.50 [552.50–733.00]	617.00 [544.00–699.00]	629.00 [562.00–718.00]	0.654

Data are presented as mean ± SD or median [Q1–Q3], or *n* (%) as appropriate, p—Kruskal–Wallis test and Pearson’s Chi-squared test for eGFR categories. Abbreviations: ALT—alanine aminotransferase; AST—aspartate aminotransferase; eGFR—estimated glomerular filtration rate; HCT—hematocrit; HDL-C—high-density lipoprotein cholesterol; HGB—hemoglobin; LDL-C—low-density lipoprotein cholesterol; PLT—platelet count; RBC—red blood cell count; SD—standard deviation; TG—triglycerides; WBC—white blood cell count.

**Table 4:** Influence of *IL-10* rs1800896 polymorphism on hematological and biochemical parameters in the entire study group.

Parameter	AA (*n* = 125)	AG (*n* = 170)	GG (*n* = 99)	*p*
Hematological parameters				
WBC (×10^9^/L)	7.68 [6.33–9.61]	7.67 [6.23–9.43]	7.71 [6.12–9.43]	0.914
RBC (×10^12^/L)	4.36 ± 0.64	4.31 ± 0.65	4.42 ± 0.51	0.383
HGB (g/dL)	12.98 ± 1.75	12.92 ± 1.95	13.44 ± 1.54	0.062
HCT (%)	38.37 ± 4.96	38.39 ± 5.49	39.45 ± 4.14	0.192
PLT (×10^9^/L)	207.00 [171.00–254.50]	203.00 [164.50–250.00]	202.50 [157.00–253.00]	0.639
Electrolytes				
Na (mmol/L)	141.00 [140.00–142.00]	141.00 [139.50–143.00]	141.00 [139.00–144.00]	0.409
K (mmol/L)	4.09 [3.79–4.39]	4.17 [3.88–4.49]	4.11 [3.87–4.40]	0.440
Kidney function				
Urea nitrogen (mg/dL)	22.25 [17.90–34.85]	27.05 [20.10–38.00]	23.15 [17.90–30.00]	0.012
Creatinine (mg/dL)	1.41 [1.09–1.79]	1.50 [1.22–1.93]	1.41 [1.17–1.77]	0.057
eGFR mL/min/1.73 m^2^	50.20 [36.02;67.10]	48.39 [37.32;57.68]	53.53 [40.95;92.25]	0.223
eGFR category, *n* (%):				0.005
<30 mL/min/1.73 m^2^	21 (16.80)	22 (12.94)	13 (13.13)	
30–59 mL/min/1.73 m^2^	57 (45.60)	114 (67.06)	58 (58.59)	
≥60 mL/min/1.73 m^2^	47 (37.60)	34 (20.00)	28 (28.28)	
Liver function				
Bilirubin (mg/dL)	0.52 [0.35–0.64]	0.49 [0.36–0.64]	0.53 [0.39–0.67]	0.624
ALT (U/L)	16.00 [12.00–20.00]	17.00 [12.00–26.00]	18.00 [13.00–26.00]	0.046
AST (U/L)	17.00 [14.00–22.00]	18.00 [15.00–23.00]	19.00 [16.00–24.00]	0.056
Lipid profile				
Total cholesterol (mg/dL)	180.00 [161.00–214.00]	185.00 [167.00–211.00]	186.00 [158.00–211.00]	0.761
HDL-C (mg/dL)	58.60 [49.55–72.80]	63.60 [46.60–75.90]	58.40 [45.10–70.85]	0.545
LDL-C (mg/dL)	90.00 [70.50–114.00]	98.00 [77.00–118.00]	92.00 [74.50–121.00]	0.398
TG (mg/dL)	135.00 [102.50–180.00]	125.00 [96.00–177.00]	126.00 [96.50–173.00]	0.632
Total lipids (mg/dL)	636.50 [535.50–721.00]	626.50 [552.00–722.00]	617.00 [551.50–688.00]	0.949

Data are presented as mean ± SD or median [Q1–Q3], or *n* (%) as appropriate, p—Kruskal–Wallis test and Pearson’s Chi-squared test for eGFR categories. Abbreviations: ALT—alanine aminotransferase; AST—aspartate aminotransferase; eGFR—estimated glomerular filtration rate; HCT—hematocrit; HDL-C—high-density lipoprotein cholesterol; HGB—hemoglobin; LDL-C—low-density lipoprotein cholesterol; PLT—platelet count; RBC—red blood cell count; SD—standard deviation; TG—triglycerides; WBC—white blood cell count.

**Table 5:** Linear regression analysis of the associations between *IL-10* rs1800896 genotype and selected laboratory parameters.

Genotypes	Model 1 Unadjusted			Model 2 Adjusted		
	Beta	95% CI	*p*-Value	Beta	95% CI	*p*-Value
Urea nitrogen (mg/dL)						
AA	—	—		—	—	
AG	1.722	−1.6, 5.0	0.304	1.441	−1.8, 4.7	0.386
GG	−0.702	−4.5, 3.1	0.714	−1.014	−4.8, 2.7	0.594
eGFR mL/min/1.73 m^2^						
AA	—	—		—	—	
AG	2.579	−3.6, 8.8	0.415	2.203	−4.0, 8.4	0.486
GG	7.595	0.50, 15	0.036	6.800	−0.29, 14	0.060
ALT (U/L)						
AA	—	—		—	—	
AG	0.588	−3.4, 4.6	0.772	0.258	−3.7, 4.2	0.898
GG	2.485	−2.1, 7.0	0.283	1.805	−2.7, 6.3	0.432

Adjusted models for age, sex, and time since transplantation. Abbreviations: ALT—alanine aminotransferase; CI—confidence interval; eGFR, estimated glomerular filtration rate.

**Table 6:** Comparison of cyclosporine (CsA) and tacrolimus (TAC) dosing according to *IL-2* rs2069762, *IL-6* rs1800795, and *IL-10* rs1800896 genotypes in the entire study group.

Parameter	Genotype 1	Genotype 2	Genotype 3	*p*
*IL-2* rs2069762	TT (*n* = 170)	TG (*n* = 180)	GG (*n* = 44)	
Patients receiving CsA, *n*	75	89	20	
CsA whole-blood concentration (ng/mL)	101.70 [78.00–138.10]	119.20 [81.00–145.20]	104.35 [75.95–130.35]	0.468
CsA daily dose (mg)	150.00 [150.00–200.00]	150.00 [150.00–200.00]	150.00 [110.00–200.00]	0.443
CsA C/D ratio	0.66 [0.49–0.90]	0.72 [0.53–0.86]	0.61 [0.46–0.96]	0.926
Patients receiving TAC, *n*	95	91	24	
TAC whole-blood concentration (ng/mL)	5.20 [4.20–7.30]	5.30 [4.20–6.95]	6.00 [4.60–8.20]	0.428
TAC daily dose (mg)	3.00 [2.00–5.00]	3.00 [2.00–4.00]	3.00 [2.00–4.00]	0.761
TAC C/D ratio	1.70 [1.16–2.46]	1.95 [1.19–3.01]	2.19 [1.34–3.08]	0.415
*IL-6* rs1800795	GG (*n* = 109)	GC (*n* = 222)	CC (*n* = 63)	
Patients receiving CsA, *n*	50	102	32	
CsA whole-blood concentration (ng/mL)	115.00 [87.30–142.30]	103.15 [74.20–138.00]	119.10 [88.30–137.95]	0.372
CsA daily dose (mg)	175.00 [150.00–200.00]	150.00 [150.00–200.00]	150.00 [150.00–200.00]	0.272
CsA C/D ratio	0.68 [0.51–0.85]	0.66 [0.50–0.88]	0.70 [0.47–0.91]	0.899
Patients receiving TAC, *n*	59	120	31	
TAC whole-blood concentration (ng/mL)	5.00 [3.90–7.00]	5.45 [4.30–7.35]	5.30 [4.20–7.30]	0.401
TAC daily dose (mg)	3.00 [2.00–4.00]	3.00 [2.00–5.00]	2.50 [2.00–4.00]	0.218
TAC C/D ratio	1.85 [1.17–2.46]	1.73 [1.16–2.77]	2.00 [1.36–3.76]	0.465
*IL-10* rs1800896	AA (*n* = 125)	AG (*n* = 170)	GG (*n* = 99)	
Patients receiving CsA, *n*	60	88	36	
CsA whole-blood concentration (ng/mL)	100.35 [81.75–141.95]	118.45 [77.05–139.15]	118.65 [79.40–145.40]	0.655
CsA daily dose (mg)	150.00 [150.00–200.00]	150.00 [150.00–200.00]	150.00 [150.00–200.00]	0.722
CsA C/D ratio	0.65 [0.50–0.90]	0.66 [0.48–0.89]	0.71 [0.52–0.85]	0.823
Patients receiving TAC, *n*	65	82	63	
TAC whole-blood concentration (ng/mL)	6.00 [4.50–7.40]	5.00 [3.90–6.90]	5.50 [4.35–7.25]	0.190
TAC daily dose (mg)	3.00 [2.00–4.00]	3.00 [2.00–5.00]	3.00 [2.00–4.25]	0.321
TAC C/D ratio	2.00 [1.40–2.95]	1.54 [1.12–2.43]	2.00 [1.12–3.21]	0.045

Data are presented as median [Q1–Q3]. Abbreviations: C/D—concentration-to-dose ratio; CsA—cyclosporine A; TAC—tacrolimus.

**Table 7:** Comparison of cyclosporine (CsA) and tacrolimus (TAC) dosing according to *IL-2* rs2069762, *IL-6* rs1800795, and *IL-10* rs1800896 genotypes stratified by sex.

Parameter	Male Genotype 1	Male Genotype 2	Male Genotype 3	*p*	FDR *p*-Value	Female Genotype 1	Female Genotype 2	Female Genotype 3	*p*	FDR *p*-Value
*IL-2* rs2069762—CsA	TT (*n* = 47)	TG (*n* = 50)	GG (*n* = 12)			TT (*n* = 28)	TG (*n* = 39)	GG (*n* = 8)		
Blood concentration (ng/mL)	99.00 [72.80–127.85]	127.05 [94.20–152.00]	101.40 [79.60–116.75]	0.014	0.252	120.65 [92.55–156.90]	103.70 [70.75–130.75]	116.65 [75.10–138.25]	0.182	0.495
Daily dose (mg)	175.00 [150.00–200.00]	200.00 [150.00–200.00]	150.00 [125.00–200.00]	0.279	0.837	150.00 [100.00–187.50]	150.00 [137.50–175.00]	150.00 [110.00–175.00]	0.816	0.864
C/D ratio	0.55 [0.43–0.71]	0.68 [0.53–0.86]	0.61 [0.40–0.83]	0.063	0.567	0.86 [0.64–1.13]	0.72 [0.50–0.88]	0.73 [0.50–1.07]	0.046	0.414
*IL-2* rs2069762—TAC	TT (*n* = 47)	TG (*n* = 59)	GG (*n* = 13)			TT (*n* = 48)	TG (*n* = 32)	GG (*n* = 11)		
Blood concentration (ng/mL)	4.90 [4.00–6.25]	5.60 [4.20–6.90]	5.90 [4.60–7.40]	0.255	0.837	5.65 [4.40–7.95]	5.05 [3.95–7.65]	6.30 [4.85–8.75]	0.568	0.730
Daily dose (mg)	3.00 [2.00–5.00]	3.00 [2.00–4.00]	3.00 [2.00–4.00]	0.853	0.950	3.00 [2.00–5.00]	2.25 [2.00–4.00]	3.00 [2.50–4.50]	0.220	0.495
C/D ratio	1.80 [1.10–2.25]	1.77 [1.17–2.65]	2.47 [1.52–2.95]	0.329	0.846	1.62 [1.17–2.58]	2.24 [1.21–3.48]	2.10 [1.07–2.98]	0.564	0.730
*IL-6* rs1800795—CsA	GG (*n* = 27)	GC (*n* = 58)	CC (*n* = 24)			GG (*n* = 23)	GC (*n* = 44)	CC (*n* = 8)		
Blood concentration (ng/mL)	109.70 [85.30–134.20]	107.35 [75.90–145.30]	119.10 [94.75–137.95]	0.844	0.950	127.00 [98.20–161.70]	103.15 [73.05–135.25]	116.05 [70.55–139.05]	0.209	0.495
Daily dose (mg)	200.00 [150.00–200.00]	162.50 [150.00–200.00]	187.50 [150.00–200.00]	0.746	0.950	150.00 [150.00–200.00]	150.00 [100.00–162.50]	150.00 [125.00–150.00]	0.156	0.495
C/D ratio	0.63 [0.49–0.74]	0.60 [0.42–0.83]	0.65 [0.47–0.84]	0.950	0.950	0.80 [0.56–1.01]	0.73 [0.56–0.98]	0.87 [0.56–0.96]	0.707	0.848
*IL-6* rs1800795—TAC	GG (*n* = 39)	GC (*n* = 66)	CC (*n* = 14)			GG (*n* = 20)	GC (*n* = 54)	CC (*n* = 17)		
Blood concentration (ng/mL)	5.20 [4.25–6.75]	5.30 [4.00–7.00]	4.75 [4.20–6.00]	0.446	0.892	4.60 [3.60–7.50]	5.95 [4.60–7.90]	5.90 [4.60–8.60]	0.162	0.495
Daily dose (mg)	3.00 [2.00–4.00]	3.00 [2.00–5.00]	3.00 [2.00–3.00]	0.397	0.892	3.00 [2.00–4.00]	3.00 [2.00–5.00]	2.00 [2.00–4.00]	0.519	0.730
C/D ratio	2.00 [1.21–2.45]	1.78 [1.14–2.65]	1.63 [1.40–2.80]	0.947	0.950	1.70 [1.16–2.49]	1.66 [1.18–2.93]	3.20 [1.33–4.30]	0.378	0.704
*IL-10* rs1800896—CsA	AA (*n* = 35)	AG (*n* = 52)	GG (*n* = 22)			AA (*n* = 25)	AG (*n* = 36)	GG (*n* = 14)		
Blood concentration (ng/mL)	99.00 [79.35–136.95]	115.75 [78.45–138.50]	114.40 [83.30–136.90]	0.710	0.950	103.70 [84.40–141.60]	119.20 [73.95–141.55]	120.05 [75.50–153.60]	0.878	0.878
Daily dose (mg)	200.00 [150.00–200.00]	187.50 [150.00–200.00]	162.50 [150.00–225.00]	0.747	0.950	150.00 [100.00–175.00]	150.00 [150.00–200.00]	135.00 [100.00–175.00]	0.391	0.704
C/D ratio	0.61 [0.49–0.77]	0.60 [0.46–0.85]	0.68 [0.53–0.79]	0.872	0.950	0.80 [0.59–1.01]	0.73 [0.55–0.90]	0.85 [0.50–1.14]	0.512	0.730
*IL-10* rs1800896—TAC	AA (*n* = 29)	AG (*n* = 49)	GG (*n* = 41)			AA (*n* = 36)	AG (*n* = 33)	GG (*n* = 22)		
Blood concentration (ng/mL)	5.20 [4.10–6.40]	5.00 [4.00–6.90]	5.20 [4.30–6.90]	0.806	0.950	6.40 [4.70–8.20]	4.70 [3.80–6.50]	6.15 [4.60–8.30]	0.037	0.414
Daily dose (mg)	3.00 [2.00–4.00]	4.00 [2.00–5.00]	3.00 [2.00–4.00]	0.169	0.761	3.00 [2.00–4.00]	3.00 [2.00–5.00]	3.50 [2.00–5.00]	0.790	0.864
C/D ratio	1.80 [1.17–2.60]	1.58 [1.20–2.10]	2.20 [1.15–3.15]	0.103	0.618	2.12 [1.49–3.20]	1.44 [1.05–2.60]	1.58 [1.08–3.23]	0.161	0.495

Data are presented as median [Q1–Q3]. Abbreviations: C/D—concentration-to-dose ratio; CsA—cyclosporine A; TAC—tacrolimus; FDR—false discovery rate.

**Table 8:** Linear regression analysis of the associations between IL-10 rs1800896 genotypes and cyclosporine (CsA) and tacrolimus (TAC) dosing.

Genotypes	Model 1 Unadjusted			Model 2 Adjusted			Model 1 Unadjusted			Model 2 Adjusted		
	Beta	95% CI	*p*-Value	Beta	95% CI	*p*-Value	Beta	95% CI	*p*-Value	Beta	95% CI	*p*-Value
CsA whole-blood concentration(ng/mL)							TAC whole-blood concentration(ng/mL)					
AA	—	—		—	—		—	—		—	—	
AG	6.882	−9.2, 23	0.400	5.617	−11, 22	0.494	−0.384	−1.3, 0.51	0.397	−0.307	−1.2, 0.58	0.494
GG	12.942	−7.3, 33	0.210	12.685	−7.6, 33	0.220	−0.016	−0.97, 0.93	0.973	0.112	−0.83, 1.1	0.815
CsA daily dose (mg)							TAC daily dose (mg)					
AA	—	—		—	—		—	—		—	—	
AG	8.864	−11, 29	0.387	6.632	−12, 26	0.490	0.667	−0.22, 1.6	0.142	0.709	−0.12, 1.5	0.095
GG	6.389	−19, 32	0.620	3.285	−20, 27	0.785	0.131	−0.82, 1.1	0.768	0.263	−0.63, 1.2	0.562
CsA C/D ratio							TAC C/D ratio					
AA	—	—		—	—		—	—		—	—	
AG	−0.019	−0.11, 0.08	0.695	−0.018	−0.11, 0.07	0.697	−0.462	−0.97, 0.04	0.072	−0.450	−0.95, 0.05	0.078
GG	0.019	−0.10, 0.14	0.744	0.031	−0.09, 0.15	0.598	0.132	−0.40, 0.67	0.628	0.121	−0.42, 0.66	0.658

Adjusted models included age, sex, time since transplantation. Abbreviations: C/D—concentration-to-dose ratio; CI—confidence interval; CsA—cyclosporine A; TAC—tacrolimus.

## Data Availability

All relevant data are included within the article.

## Supplementary Materials

The following supporting information can be downloaded at: https://www.mdpi.com/article/10.3390/biomedicines14081858/s1, Table S1: General characteristics of patients after kidney transplantation; Table S2: Characteristics of laboratory data of renal transplant patients treated with immunosuppressive therapy; Table S3: Characteristics of laboratory data in the entire group of patients and divided by age of patients; Table S4: Characteristics of laboratory data according to time since transplantation; Table S5: Distribution of IL-2 genotypes and genetic model–based association with CsA and TAC treatment groups; Table S6: Distribution of IL-6 genotypes and genetic model–based association with CsA and TAC treatment groups; Table S7: Distribution of IL-10 genotypes and genetic model–based association with CsA and TAC treatment groups.

## Abbreviations

The following abbreviations are used in this manuscript:

ALT	alanine aminotransferase
AST	aspartate aminotransferase
BMI	body mass index
CsA	cyclosporine
eGFR	estimated glomerular filtration rate
HCT	hematocrit
HDL-C	high-density lipoprotein cholesterol
HGB	hemoglobin
LDL-C	low-density lipoprotein cholesterol
PLT	platelet count
RBC	red blood cell count
TAC	tacrolimus
TG	triglycerides
WBC	white blood cell count
