# Supplementary material for: Associations of IL-2, IL-6 and IL-10 Gene Polymorphisms with Biochemical Parameters and Calcineurin Inhibitor Dosing in Kidney Transplant Recipients
Source: Biomedicines. 2026 Aug 18;14(8):1858. doi: 10.3390/biomedicines14081858 (PMC13509670; doi:10.3390/biomedicines14081858)
Supplement: Supplementary file 1 [file biomedicines-14-01858-s001.zip › biomedicines-4474624-supplementary.pdf]

Supplementary Materials

Table S1. General characteristics of patients after kidney transplantation

Parameter	*Total group N=394*	*Women (W) n=166*	*Men (M) n=228*	*p (W) vs. (M)*
Age (years)	52.36 ± 13.29	52.22 ± 12.86	52.45 ± 13.62	0.866
≤50	158 (40.10%)	68 (40.96%)	90 (39.47%)	0.947
51-59	100 (25.38%)	41 (24.70%)	59 (25.88%)	
≥60	136 (34.52%)	57 (34.34%)	79 (34.65%)	
Blood pressure (mmHg)				
systolic	132.39 ± 12.25	131.52 ± 12.66	133.03 ± 11.94	0.228
diastolic	82.66 ± 7.85	81.55 ± 8.09	83.46 ± 7.59	0.017
BMI (kg/m^2)	26.75 ± 4.95	26.37 ± 5.02	27.02 ± 4.89	0.201
<18.5 underweight	5 (1.27%)	2 (1.20%)	3 (1.32%)	0.107
18.5 – 24.99 normal weight	145 (36.80%)	70 (42.17%)	75 (32.89%)	
25 – 29.99 overweight	158 (40.10%)	55 (33.13%)	103 (45.18%)	
≥30 obesity	86 (21.83%)	39 (23.49%)	47 (20.61%)	

BMI – body mass index

Table S2. Characteristics of laboratory data of renal transplant patients treated with immunosuppressive therapy

Parameter	*Total group N=394*	*Women (W) n=166*	*Men (M) n=228*	*p (W) vs. (M)*
WBC (10^9/L)	7.69 [6.25;9.54]	7.47 [6.04;9.46]	7.79 [6.33;9.56]	0.186
RBC (10^{12}/L)	4.36 ± 0.61	4.15 ± 0.57	4.50 ± 0.61	<0.001
HGB (g/dL)	13.07 ± 1.80	12.41 ± 1.62	13.55 ± 1.78	<0.001
HCT (%)	38.65 ± 5.02	36.88 ± 4.57	39.92 ± 4.96	<0.001
PLT (10^9/L)	204.00 [164.00;253.00]	217.00 [180.00;268.50]	195.00 [159.00;242.50]	<0.001
Na (mmol/L)	141.00 [139.00;143.00]	141.00 [139.00;142.70]	141.00 [140.00;144.00]	0.067
K (mmol/L)	4.14 [3.84;4.43]	4.09 [3.80;4.38]	4.20 [3.87;4.50]	0.024
Bilirubin(mg/dL)	0.50 [0.37;0.65]	0.46 [0.33;0.60]	0.52 [0.39;0.68]	0.004
Urea nitrogen (mg/dL)	24.95 [18.95;34.90]	23.70 [17.30;32.10]	25.80 [20.00;35.40]	0.014
Creatinine (mg/dL)	1.44 [1.17;1.84]	1.27 [1.05;1.68]	1.58 [1.27;1.96]	<0.001
eGFR (ml/min/1.73m^2)				0.252
<30	56 (14.21%)	29 (17.47%)	27 (11.84%)	
30-59	229 (58.12%)	95 (57.23%)	134 (58.77%)	
>60	109 (27.66%)	42 (25.30%)	67 (29.39%)	
ALT (U/L)	17.00 [12.00;24.00]	14.00 [10.00;19.00]	18.00 [14.00;27.00]	<0.001

AST (U/L)	18.00 [15.00;23.00]	17.00 [14.00;22.00]	19.00 [16.00;24.00]	0.050
Total cholesterol (mg/dL)	185.00 [161.50;212.50]	187.50 [165.00;216.00]	182.00 [161.00;208.00]	0.197
HDL-C (mg/dL)	60.20 [46.60;73.90]	65.60 [54.50;76.60]	58.15 [42.35;68.15]	<0.001
LDL-C (mg/dL)	94.00 [74.00;118.00]	91.50 [72.00;118.00]	96.00 [75.00;117.50]	0.716
TG (mg/dL)	131.00 [97.00;178.00]	131.00 [98.00;172.00]	130.50 [97.00;185.50]	0.689
Total lipids (mg/dL)	625.00 [549.00;715.00]	648.00 [553.00;729.00]	618.00 [547.00;711.50]	0.457

ALT - alanine transaminase, AST - aspartate transaminase, eGFR - estimated glomerular filtration rate, HCT – hematocrit, HDL-C – high-density lipoprotein cholesterol; HGB – hemoglobin, K- potassium, LDL-C – low-density lipoprotein cholesterol; Na – sodium, PLT – blood platelets, RBC – red blood cells, TG – triglycerides, WBC – white blood cells

Table S3. Characteristics of laboratory data in the entire group of patients and divided by age of patients.

Parameter	*≤50 years (n=158)*	*51-59 years (n=100)*	*≥60 years (n=136)*	*p*
WBC (10^9/L)	8.29 [6.75;10.19]	7.46 [5.95;9.30]	7.20 [5.85;8.73]	<0.001
RBC (10^{12}/L)	4.39 ± 0.59	4.30 ± 0.58	4.36 ± 0.67	0.495
HGB (g/dL)	13.04 ± 1.83	13.04 ± 1.60	13.13 ± 1.92	0.906
HCT (%)	38.60 ± 4.88	38.48 ± 4.67	38.83 ± 5.44	0.861
PLT (10^9/L)	217.00 [173.50;265.50]	205.00 [167.50;254.00]	195.50 [158.00;246.00]	0.017
Na (mmol/L)	141.00 [140.00;142.00]	141.00 [139.00;143.00]	141.50 [140.00;144.00]	0.339
K (mmol/L)	4.08 [3.83;4.36]	4.17 [3.88;4.46]	4.20 [3.83;4.50]	0.241
Bilirubin (mg/dL)	0.41 [0.31;0.56]	0.52 [0.40;0.68]	0.57 [0.42;0.72]	<0.001
Urea nitrogen (mg/dL)	23.80 [16.80;34.50]	26.05 [21.15;34.90]	25.30 [18.85;35.20]	0.078
Creatinine (mg/dL)	1.48 [1.15;1.98]	1.48 [1.23;1.83]	1.38 [1.16;1.74]	0.183
eGFR (ml/min/1.73m^2)<br><30<br>30-59<br>>60	 26 (16.46%)<br>80 (50.63%)<br>52 (32.91%)	 16 (16.00%)<br>63 (63.00%)<br>21 (21.00%)	 14 (10.29%)<br>86 (63.24%)<br>36 (26.47%)	0.090
ALT (U/L)	18.00 [12.00;26.00]	17.00 [12.00;24.00]	16.00 [12.00;22.00]	0.571
AST (U/L)	18.00 [14.00;23.00]	19.00 [15.50;22.50]	18.00 [15.50;22.00]	0.763
Total cholesterol (mg/dL)	189.00 [166.50;214.00]	189.50 [168.00;214.00]	179.00 [158.00;203.00]	0.059
HDL-C (mg/dL)	59.10 [45.30;68.70]	59.00 [50.90;71.90]	64.60 [46.70;78.20]	0.185
LDL-C (mg/dL)	99.00 [76.00;120.00]	98.00 [78.50;119.00]	84.00 [68.00;106.00]	0.031
TG (mg/dL)	137.00 [98.50;180.50]	137.00 [104.00;186.00]	119.00 [90.00;161.00]	0.076
Total lipids (mg/dL)	645.00 [558.00;740.00]	641.00 [564.00;725.00]	607.00 [528.00;668.00]	0.027

ALT - alanine transaminase, AST - aspartate transaminase, eGFR - estimated glomerular filtration rate, HCT – hematocrit, HDL-C – high-density lipoprotein cholesterol; HGB – hemoglobin, K- potassium, LDL-C – low-density lipoprotein cholesterol; Na – sodium, PLT – blood platelets, RBC – red blood cells, TG – triglycerides, WBC – white blood cells

Table S4. Characteristics of laboratory data according to time since transplantation.

Parameter	*<7 years (n=179)*	*≥7 years (n=215)*	*p*
WBC (10^9/L)	7.73 [6.33;9.47]	7.62 [6.21;9.55]	0.410
RBC (10^{12}/L)	4.37 ± 0.61	4.34 ± 0.62	0.691
HGB (g/dL)	13.06 ± 1.87	13.08 ± 1.75	0.919
HCT (%)	38.61 ± 5.19	38.68 ± 4.88	0.887
PLT (10^9/L)	197.50 [160.00;245.00]	212.50 [169.50;255.50]	0.060
Na (mmol/L)	141.00 [140.00;143.00]	141.00 [139.00;143.00]	0.721
K (mmol/L)	4.13 [3.82;4.46]	4.15 [3.84;4.41]	0.895
Bilirubin (mg/dL)	0.46 [0.35;0.66]	0.52 [0.39;0.65]	0.092
Urea nitrogen (mg/dL)	23.30 [17.90;31.40]	26.20 [20.10;35.40]	0.012
Creatinine (mg/dL)	1.40 [1.16;1.81]	1.50 [1.19;1.90]	0.247
eGFR (ml/min/1.73m^2)			0.720
<30	23 (12.85%)	33 (15.35%)	
30-59	104 (58.10%)	125 (58.14%)	
>60	52 (29.05%)	57 (26.51%)	
ALT (U/L)	18.00 [12.00;25.00]	16.00 [12.00;22.00]	0.140
AST (U/L)	18.00 [14.00;21.00]	19.00 [16.00;24.00]	0.009
Total cholesterol (mg/dL)	185.50 [161.00;212.00]	185.00 [164.50;214.00]	0.756
HDL-C (mg/dL)	60.15 [46.40;71.60]	60.70 [46.60;75.90]	0.640
LDL-C(mg/dL)	92.00 [73.50;120.50]	97.00 [74.50;116.50]	0.905
TG (mg/dL)	138.50 [102.50;180.00]	125.00 [94.00;176.00]	0.152
Total lipids (mg/dL)	642.00 [549.00;734.50]	623.00 [549.00;696.00]	0.425

ALT - alanine transaminase, AST - aspartate transaminase, eGFR - estimated glomerular filtration rate, HCT – hematocrit, HDL-C – high-density lipoprotein cholesterol; HGB – hemoglobin, K- potassium, LDL-C – low-density lipoprotein cholesterol; Na – sodium, PLT – blood platelets, RBC – red blood cells, TG – triglycerides, WBC – white blood cells

Table S5. Distribution of *IL-2* genotypes and genetic model–based association with CsA and TAC treatment groups.

IL-2 rs2069762	*CsA (n=184)*	*TAC (n=210)*	*OR (95%CI)*	*p*	*AIC*
		Codominant model			
TT	75 (40.8)	95 (45.2)	Ref.	0.598	549.5
TG	89 (48.4)	91 (43.3)	0.81 (0.53-1.23)		
GG	20 (10.9)	24 (11.4)	0.95 (0.49-1.84)		
		Dominant model			
TG+GG	109 (59.2)	115 (54.8)	0.83 (0.56-1.24)	0.371	547.7
		Recessive model			
TT+TG	164 (89.1)	186 (88.6)	1.06 (0.56-1.99)	0.860	548.5
		Overdominant model			
TT+GG	95 (51.6)	119 (56.7)	0.82 (0.55-1.21)	0.317	547.5
		Additive model			
0.1.2	184 (46.7)	210 (53.3)	0.91 (0.68-1.23)	0.559	548.1

Data are presented as numbers (percentages). Odds ratios (ORs) with 95% confidence intervals (CIs) were calculated using logistic regression. The TT genotype was used as the reference category. AIC, Akaike Information Criterion.

Table S6. Distribution of *IL-6* genotypes and genetic model–based association with CsA and TAC treatment groups.

IL-6 rs1800795	*CsA (n=184)*	*TAC (n=210)*	*OR (95%CI)*	**p**	**AIC**
		Codominant model			
GG	50 (27.2)	59 (28.1)	Ref.	0.777	550.0
GC	102 (55.4)	120 (57.1)	1.00 (0.63-1.58)		
CC	32 (17.4)	31 (14.8)	0.82 (0.44-1.53)		
		Dominant model			
GC+CC	134 (72.8)	151 (71.9)	0.95 (0.61-1.49)	0.838	548.4
		Recessive model			
GG+GC	152 (82.6)	179 (85.2)	0.82 (0.48-1.41)	0.478	548.0
		Overdominant model			
GG+CC	82 (44.6)	90 (42.9)	1.07 (0.72-1.6)	0.733	548.4
		Additive model			
0.1.2	184 (46.7)	210 (53.3)	0.92 (0.68-1.25)	0.589	548.2

Data are presented as numbers (percentages). Odds ratios (ORs) with 95% confidence intervals (CIs) were calculated using logistic regression. The GG genotype was used as the reference category. AIC, Akaike Information Criterion.

Table S7. Distribution of *IL-10* genotypes and genetic model–based association with CsA and TAC treatment groups.

IL-10 rs1800896	*CsA (n=184)*	*TAC (n=210)*	*OR (95%CI)*	*p*	*AIC*
Codominant model					
AA	60 (32.6)	65 (31.0)	Ref.	0.046	544.3
AG	88 (47.8)	82 (39.0)	0.86 (0.54-1.37)		
GG	36 (19.6)	63 (30.0)	1.62 (0.94-2.77)		
Dominant model					
AG+GG	124 (67.4)	145 (69.0)	1.08 (0.71-1.65)	0.725	548.4
Recessive model					
AA+AG	148 (80.4)	147 (70.0)	1.76 (1.10-2.82)	0.017	542.7
Overdominant model					
AA+GG	96 (52.2)	128 (61.0)	0.70 (0.47-1.04)	0.079	545.4
Additive model					
0.1.2	184 (46.7)	210 (53.3)	1.24 (0.95-1.62)	0.110	545.9

Data are presented as number (percentage). Odds ratios (ORs) with 95% confidence intervals (CIs) were calculated using logistic regression. The AA genotype was used as the reference category. AIC, Akaike Information Criterion.
